# Analysis of Voluntary Environmental Behavior and Innovation Quality of Chinese Textile Enterprises Based on Grounded Theory and Propensity Score Matching

**DOI:** 10.1155/2022/9736667

**Published:** 2022-08-09

**Authors:** Yabin Yu, Hua Cheng, Qian Xu

**Affiliations:** ^1^School of Economics and Management, Zhejiang Sci-Tech University, Hangzhou 310000, China; ^2^College of Economics & Management, China Jiliang University, Hangzhou 310018, China

## Abstract

At a new stage of “carbon peak” and “carbon neutral,” we need to strengthen the internal impetus of institutions and policies to promote ecological civilization construction. The initiative implementation of voluntary environmental behavior (VEB) is an effective way for enterprises to achieve high quality sustainable development. The VEB of enterprises include but are not limited to compliance with voluntary environmental regulations (VER). Different from the existing literature on enterprise VER, most of which are just the verification of ISO14001, the study conducts a comprehensive exploratory research on the theoretical framework of what is driving the VEB of Chinese textile enterprises. First, based on grounded theory, the paper coded the hand-collected environmental text information of Chinese listed textile enterprises from 2004 to 2018 and constructed a complete theoretical analysis framework from motivation to performance evaluation of enterprise VEB. Second, PSM method is taken to verify the conceptual model. As a result, we find the following: (1) in addition to ISO14001, the textile enterprises involve many industry-specific VER. (2) The VEB and VER of Chinese textile enterprises promote each other. (3) Either following VER or implementing VEB can effectively increase the innovation input and then actively improve the innovation quality of Chinese textile enterprises. In this way, the conclusion not only provides analysis ideas for future empirical research, but also offers practical suggestions to Chinese textile enterprises and policy makers for environmental management.

## 1. Introduction

Environmental protection is China's basic state policy, and sustainable development strategy is a national strategy. After setting the goal of “carbon peak” and “carbon neutral,” we need to strengthen the internal impetus of institutions and policies to promote ecological civilization to a new stage of more conscious and proactive construction. With global warming and dwindling resources, every member of the planet has a responsibility to contribute to a better tomorrow. Both individuals and social organizations should take an active part in environmental protection. The research on environmental behaviors of enterprises should not be limited to traditional command-based and market-based environmental regulations, but increase the research on voluntary environmental regulations (VER) [1], which can better describe the enterprise's willingness to protect the environment. Since it is different from the traditional command-based and market-based environmental regulations, the VER is not a mandatory regulation, and enterprises can choose according to their environmental intentions. However, the voluntary environmental behavior (VEB) of enterprises is not limited to following the VER, but including multilevels of enterprise activities related environment protection. The concept of enterprise VEB is broader and worthy of attention, becoming the theme of the study.

When we review the existing literature, most studies on VEB and VER are conducted from two perspectives. One is by obtaining data through questionnaire survey to study the VEB of enterprise employees [2–4]; the other is to study the relatively common VER ISO14001 [5, 6]. Besides, by comparing domestic and foreign literature, it can be found that there is a lack of research on VEB and VER under China's socioeconomic background. It might mainly be because command-based environmental regulations have been dominated for a long time in China, and the practice of VER is relatively insufficient [7], which provide full space for the new study of VEB and VER.

Therefore, the innovation of this paper lies in the construction of a widely applicable theoretical framework of enterprise VEB based on grounded theory, which provides analysis ideas for future empirical research. In addition, under the background that China's economy is gradually changing from an export-oriented development mode to a domestic cycle, the living environment of traditional manufacturing industry is also changing. Among many traditional manufacturing enterprises, textile enterprises are representative. Today, China has become a textile power. Textile enterprise is vital in the national economy. The environmental problem is an important challenge that traditional textile enterprises have to be faced with [8]. In this way, it is of great practical significance to study whether traditional textile enterprises can make full use of intelligent method and voluntarily carry out environmentally friendly behaviors to support the sustainable development of themselves and the society.

But in practice, what kind of ways do enterprises manage to protect the environment? Is it through indirect behavior at the individual level or direct behavior at the enterprise level? Is it the compulsion of external policy, or the spontaneous behavior of the enterprise? Traditional command-based policies mostly punish those who fail to meet the standards after the event, but do not encourage environmental pioneers [9]. However, sometimes enterprises may only follow in name but do not carry out substantive green management [10, 11]. We can further classify the relevant literature into the following two categories.

### 1.1. VEB

Most studies on VEB focus on employee behavior. Employees' cognition of corporate social responsibility can effectively promote their VEB, which can be promoted by substantive incentives but can be weakened by symbolic incentives. It also requires enterprises to take social responsibility into consideration when formulating sustainable development strategies, so as to truly promote employees' VEB [2]. At the same time, how employees participate in VEB is also affected by the organization's definition of the concept [3]. At the same time, individual consumption reduction is also one of the behaviors to achieve sustainable economy [12].

From the perspective of enterprises, data from Japanese manufacturing enterprises show that enterprises' spontaneous application of environmental management system can not only effectively reduce the negative impact on the environment, but also effectively improve the productivity of enterprises [13]. At the same time, corporate environmentalism can effectively promote enterprise innovation [14]. In terms of environmental information disclosure, it may only be reflected in quantity, but the quality is not satisfactory [15]. However, the disclosure of voluntary environmental information does affect the future value of enterprises [16].

### 1.2. VER

Even VER cannot be implemented without the promotion of government departments [17], and the existence of government is conducive to the construction of green cooperation network for private enterprises [18]. The experience from India shows that the willingness of enterprises to comply with VER will be affected by enterprises' size, location, export-orientation, intangible asset value, and other factors [19]. Moreover, voluntary compliance with environmental regulations does help enterprises to improve the environmental performance [20].

As a VER tool, ISO14001 is a supplement to the traditional environmental regulations. Research shows that ISO14001 certification can significantly promote enterprise innovation [21], so managements should take ISO14001 certification as a core of the company's innovation development strategy, effectively carrying out environmental management activities and investing resources in a long-term way [5]. In addition, the implementation of voluntary environmental management systems (such as ISO14001 and United Nations Global Compact) by enterprises can effectively improve their environmental performance [22]. At the same time, green competitors and external environment are also important factors affecting whether an enterprise complies with VER [23]: the more stringent the formal environmental regulations and the more ISO14001 certified enterprises in the region, the more likely an enterprise is to carry out ISO14001. The enterprise with high degree of external and ISO9000 certification is more likely to carry out ISO14001 [6]. However, the communication of positive image may not be one of the motivations for enterprises to follow VER [24], but the management diversity may be one of the influencing factors [25].

In general, we find that the previous literature of the theme is centered on employees' voluntary behavior and ISO14001, the data source is mainly questionnaire and enterprise financial report data, and for the method most research applies statistical analysis [2–4] and some adopt social network [18]. However, most of the existing studies are only test static single data, which cannot fully reflect the whole VEB of enterprises. Therefore, this research firstly applies the grounded theory to comprehensively and systematically characterize the VEB of Chinese textile enterprises through the three-level coding technology and then takes PSM method to further verify the conceptual model. To make up the existing research deficiencies from the theme, research methods and data are used.

## 2. Material and Methods

### 2.1. Research Method

In order to fully characterize the VEB of textile enterprises in China, the grounded theory is adopted in the paper. Grounded theory is a classical method widely used in qualitative research. Through repeated reading and comparison of text information, effective information points are naturally presented, so as to conceptualize behavior patterns, extract core elements, and describe the logical relationship between core elements [26].

At the same time, in order to verify the rationality of the model, we take Propensity Score Matching (PSM) method after model construction. The variable included and research framework are introduced in Sections 2.4 and 2.5.

### 2.2. Data Sources

In the paper, 39 enterprises with sufficient environmental text information were selected from 111 listed textile companies in China, and more than 165,395 words of environment-related text information were hand-extracted from their annual report and social responsibility report from 2004 to 2018. Among them, the sample size with valid text information sample reached 218.

### 2.3. Data Processing

After collecting and sorting the environmental text of the sample enterprises, we mainly combed it manually and analyzed the data with the help of NVivo12. 30 enterprises coded to build models, and the remaining 9 were used for saturation test. Due to different data sources, sometimes text information and report information do not match, and repeated comparison and cross-data verification are required.

### 2.4. Variables

#### 2.4.1. Dependent Variables

Innovation input: the input of innovation activities mainly considers the enterprise's innovation capital input (calculated by the enterprise's R&D expenditure) [27, 28].

Innovation quality: the paper mainly studies the impact of environmental regulation on innovation quality of textile enterprises, and greenness is an important aspect of innovation quality. Therefore, we use green innovation to measure the innovation quality here, and green patent is a common indicator to measure the performance of green innovation [8].

#### 2.4.2. Independent Variable

VEB: the VEB data are obtained from the grounded theory research conclusion in the above part. Textile enterprises that have implemented VEB are marked as 1, while those that have not implemented VEB are marked as 0.

VER: the most widely practiced VER in China is ISO14001 [7]. The study measures the compliance of enterprises with VER based on whether enterprises have obtained ISO14001 certification. Textile enterprises that have completed ISO14001 will be recorded as 1, and those that have not completed ISO14001 will be recorded as 0.

#### 2.4.3. Control Variables

Considering the influence of enterprise heterogeneity, the subindustry, region, and property rights of the enterprise are put into the model as control variables [8]. In addition, the size and operating performance of an enterprise also affect the innovation behavior of an enterprise, the size (measured as total assets), revenue growth rate, and return on total assets (ROA) [28].

### 2.5. Counterfactual Research Framework

We take the textile enterprises implemented VEB/VER as treatment group, and the other ones as control group. Since it is difficult to ensure that the probability distribution of the experimental group and the control group is consistent, the counterfactual research framework is constructed by referring to Rosenbaum and Rubin's method [29], to find a control group as similar as possible to the experimental group, so as to effectively reduce the biased estimation caused by the nonrandom distribution of samples. In this context, the average treatment effect (ATT) of the treatment experimental group (textile enterprises implementing VEB/VER) was defined as

(1) $$\begin{array}{c} ATT=E\left(Y1|M=1\right)-E\left(Y0|M=1\right)=EE\left(Y1-Y0|M=1\right), \end{array}$$

where *Y*1 is the input/output level of textile enterprises' innovation activities when they implement VEB/VER, and Y0 is the input/output level of textile enterprises' innovation activities when they do not implement VEB/VER. The premise hypothesis of ATT is that when the textile enterprise implements VEB/VER (*M* = 1), the difference (*Y*1–*Y*0) of the input/output level of textile enterprise innovation activities from those enterprises without implementing VEB/VER is calculated. In this way, the influence of implementing VEB/VER on the input/output of innovation activities of textile enterprises can be obtained. However, in the model (1), the result of *E* (*Y*1|*M* = 1) can be observed, but *E* (*Y*0 |*M* = 1) cannot, called counterfactual result. Therefore, we can use PSM method to construct the alternative indicator of *E* (*Y*0|*M* = 1). In other words, on the premise of a set of covariables, a virtual sample group (control group) matching with the textile enterprises implementing VEB/VER (treatment group) was constructed by PSM method, so as to establish a reasonable counterfactual framework.

## 3. Results and Discussion

### 3.1. Descriptive Statistics

**Figure 1:**
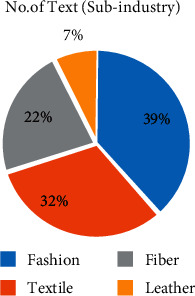
Number of texts by subindustry.

**Figure 2:**
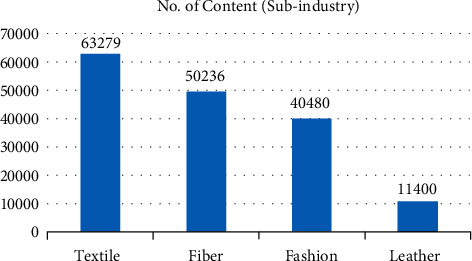
Number of contents by subindustry.

Based on the statistics of environmental disclosure information of sample enterprises, we analyze the distribution of samples (number of texts, how many texts of sample enterprise disclose environment protection information) and content (number of contents, how many words of the sample enterprise disclose environment protection information) from three dimensions: subindustry, property right, and region of enterprises. First of all, for the number of texts (Figure 1), the clothing industry accounts for the highest proportion, even higher than the textile and fiber industries which are traditionally considered to have more serious environmental problems. Due to the small sample size of the leather industry, it is normal that it ranks last. However, digging into the content of disclosed information (Figure 2), it is true that the textile industry and fiber industry are more specific and in depth in green management. Because of relatively less pollution, the clothing industry has more green management from the overall strategy, specifically from the daily office behavior to implementing environmental protection.

**Figure 3:**
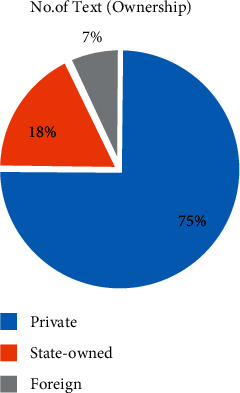
Number of texts by ownership.

**Figure 4:**
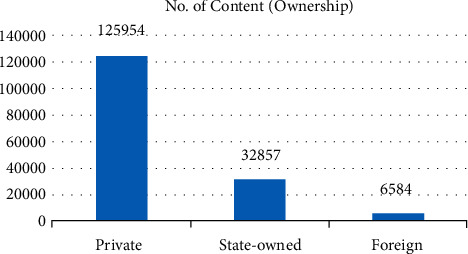
Number of contents by ownership.

Secondly, from the perspective of the property rights of enterprises, private enterprises have an absolute advantage in both the sample number (Figure 3) and the content (Figure 4), which is inseparable from the fact that private enterprises account for 78% of listed textile enterprises in China.

**Figure 5:**
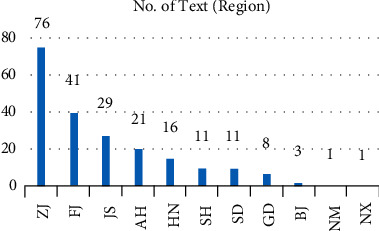
Number of texts by region.

**Figure 6:**
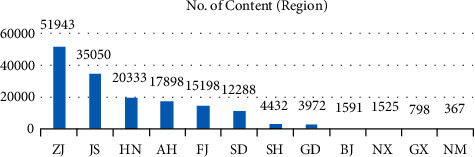
Number of contents by region.

In addition, from the regional point of view, the environmental information disclosure of Zhejiang province (ZJ) ranks first in both sample size (Figure 5) and content (Figure 6) and is far ahead of other provinces. On the one hand, Zhejiang province (ZJ) itself is a big textile province of China, and the listed textile enterprises in Zhejiang province (ZJ) account for 27.93% of all listed textile enterprises in China. On the other hand, in the 31 listed textile enterprises in Zhejiang province (ZJ), 15 have disclosed environmental text information, accounting for nearly half. Jiangsu Province (JS), which ranks second in terms of content and information disclosure and belongs to the Yangtze River Delta region with Zhejiang province (ZJ), is also a big textile province of China and also takes the lead in green management.

### 3.2. Modeling

#### 3.2.1. Open Coding

Open coding is a dialogue between researcher and data and the process of continuous decomposition and analysis of the original data. Through repeated comparison of the similarities and differences of the original data word by word, objects with similar characteristics are given the same conceptual label [30]. By sorting out the obtained environmental text information of China's listed textile enterprises, we screened and obtained 5285 words of key coding statements from the 165,395 words of original data and further summarized the following categories (Table 1).

#### 3.2.2. Axial Coding

The axial coding is to construct the master-slave category of the concepts formed by the open coding, and the combining of the structure between concepts is another test of data [30]. We further analyze the 16 categories obtained by open coding to explore the causal and subordinate relationships between different categories. In this stage of coding, the 16 parallel categories formed by open coding are further classified into five main categories (external factor, internal factor, VEB, VER, performance). The relationship between categories is shown in Table 2.

#### 3.2.3. Selective Coding

Selective coding is to find the “core category” of the study and build a story line around it for description [30]. Through further comparative analysis of 16 concepts obtained by open coding and 5 main categories formed by axial coding, the core category “VEB” that can radiate the whole story is found out. And the category connecting its upstream and downstream is thus divergent to form a complete logical chain. The complete logical chain can help us form a theoretical framework for the analysis of enterprises' VEB, as shown in Table 3.

### 3.3. Theoretical Saturation Test

Theoretical saturation test means that all categories are fully reflected in their attributes, dimensions, and forms of change, and further data collection and analysis will hardly make new contributions to the conceptualization. The paper coded the text information of the remaining 9 sample enterprises but failed to get the initial concept and relationship structure significantly different from the above 16 categories and 5 main categories, proving that the information of this theoretical research has reached saturation, and further information collection and sorting can be stopped.

### 3.4. Theoretical Model Construction and Interpretation

**Figure 7:**
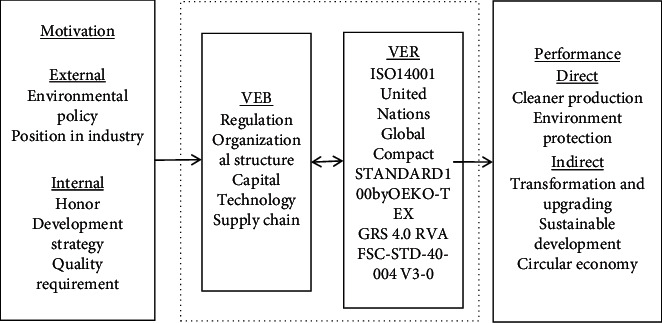
Theoretical framework for the motivation and influence path of VEB/VER.

Through open coding, axial coding, and selective coding, the following theoretical framework (Figure 7) is formed for the motivation and influence path of the VEB of Chinese textile enterprises.

#### 3.4.1. Motivation

From the external perspective, first of all, environmental policy is a very important influencing factor. No matter the national environmental protection law, environmental protection tax law, or the local water pollution prevention and control regulations, river chief system will restrict the implementation of VEB of enterprises, which is also the macroenvironmental motivation in the process of enterprise development. Secondly, the position of enterprises in the industry will also have an impact on the VEB of enterprises. If an enterprise is in a leading position in its industry, it is more motivated to lead the industry in environmental protection, such as becoming the first enterprise in the industry to conduct a voluntary environmental certification or being one of the few enterprises in the industry to produce a certain environmentally friendly product.

From the internal point of view, the honorary titles such as “energy-saving advanced enterprise” and “regional green enterprise” help enterprises to further implement VEB. And if companies put environmental protection into their development strategies, it will also be implemented in the daily VEB. The pursuit of excellence of some advanced enterprises will also make them not satisfied with merely reaching the national environmental protection standards, but through increasing investment in capital and technology to achieve excellence in environmental protection.

External motivation and internal motivation do not affect the VEB of enterprises alone but also have a mixed effect. Because both external and internal factors jointly constitute the macro-, medium, and micro environment for Chinese textile enterprises to implement VEB and follow VER, there are cross-layer interactive influences.

#### 3.4.2. VEB vs. VER

VEB is a series of actions taken by enterprises to achieve the purpose of environmental protection, including but not limited to the formulation of internal environmental regulations, the establishment of the corresponding environmental management organization, increasing capital investment, research and development of environmental protection technology, and the implementation of supply chain management, etc.

VER is a series of environmental protection standards that enterprises voluntarily follow. The most representative one is ISO14001 as an environmental management system certification for the whole enterprise to be environment friendly. In addition, it also includes ISO50001 energy system certification for energy conservation, voluntary environmental protection product certification, and standard 100BYOEKo-TEX certification (one of the most well-known labels in the world stating that textiles and accessories do not pose a health risk), etc.

**Figure 8:**
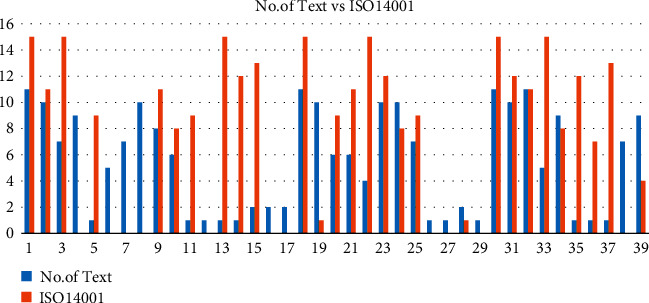
Number of text vs. ISO14001.

In order to analyze the relationship between Chinese textile enterprises' implementation of VEB and compliance with VER, software STATA was used to analyze the correlation between the sample size of Chinese text information (number of text) and the number of ISO14001 certifications. It can be seen from the results in Table 4 that there is a positive correlation between the two. In Figure 8, the comparative data of the number of text disclosure of sample enterprises and their ISO14001 certification can also be further supported. And theoretically, the two are complementary [31]. Textile enterprises that carry out substantive green management disclose more text information and are more inclined to carry out ISO14001 certification. In turn, textile enterprises with ISO14001 certification will be more standardized to implement VEB and information disclosure.

#### 3.4.3. Performance

Whether an enterprise implements VEB or complies with VER, the effectiveness will be reflected in its performance, which can be divided into direct performance and indirect performance.

The direct performance of enterprises' VEB must be a breakthrough in environmental protection. Enterprises can achieve the cleaner production through investment in R&D and technological transformation. In this way, the direct purpose of environmental protection can be achieved from the production process and results.

The indirect performance of enterprises' VEB is mainly reflected in the economic benefits of long-term development, such as enabling China's textile enterprises to achieve transformation and upgrading, sustainable development, and circular economy. The VEB of enterprises can help to get more competitive and long-term development in the future, which are also in line with the expectations of “Porter hypothesis.”

### 3.5. Propensity Score Matching Method

Since it is “voluntary environmental behavior” and “voluntary environmental regulation,” the implementation of enterprises has a certain “self-choice.” In order to further verify the rationality of the model proposed by the grounded theory, predicting the innovation quality impact of enterprise after “voluntary” implementation, the nonparametric estimation method, Propensity Score Matching (PSM) method, will be introduced. Under the premise that the covariables of the two sample firms are as similar or the same as possible, the propensity score of each sample firm to enter the treatment group is calculated. This method can accurately evaluate the effect of implementing VEB/VER on the input/output level of textile enterprises' innovation activities.

#### 3.5.1. The Effect of VER on Innovation Input of Chinese Textile Enterprises

Firstly, the samples of the treatment group and the control group were matched with *k*-neighborhood 1:4 within the caliper (default radius 0.05) to study the effect of VER on innovation input of Chinese textile enterprises.

**Figure 9:**
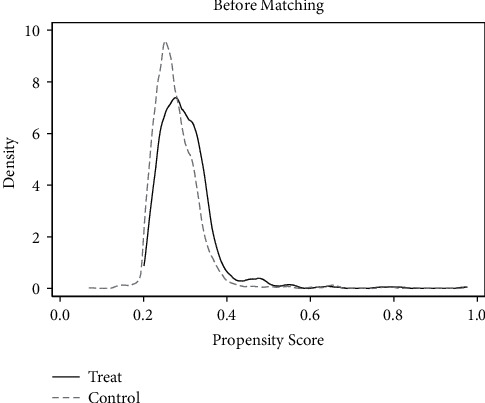
Density function before matching.

**Figure 10:**
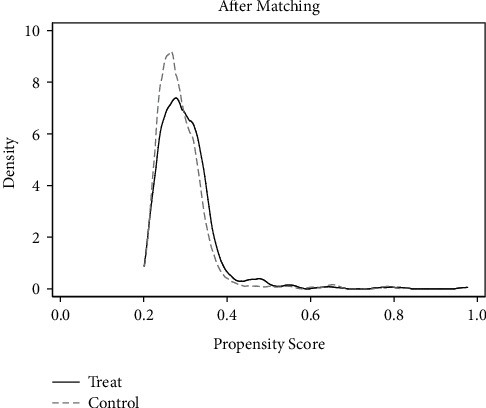
Density function after matching.

*(1) Common Support Area and Balance Check*. According to the matching results in Table 5, only 1 sample was lost and 1664 samples were retained in the treatment group and the control group. Most of the observed values were within the common value range, as can be seen from Figures 9 and 10, indicating good matching effect.

**Figure 11:**
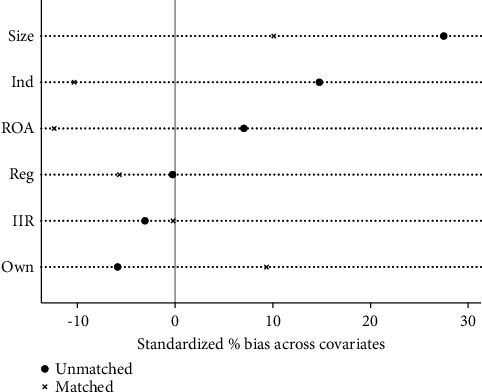
Matching validity test.

In order to ensure the reliability of PSM, it is necessary to test the balance of covariables to ensure that there are no significant differences in other influencing factors except VER. The results in Table 6 show that, compared with the results before matching, the standardization bias (% bias) of most covariables after matching is basically controlled below 10%, which reduces the total matching bias to a large extent. Although the standardized deviation of ROA is -12.4% after matching, it is still within the acceptable range. The results show that the matching of covariables is reliable and has passed the balance test.  Figure 11 intuitively reflects the improvement of bias after sample matching.

*(2) Measurement of Treatment Effects*. According to Model 1, the impact of VER on innovation input of textile enterprises is measured. It can be seen from Table 7 that implementing VER can effectively promote innovation input of textile enterprises (the difference of ATT is positive and t-stat is 3.21).

#### 3.5.2. The Effect of VER/VEB on Innovation Quality of Textile Enterprises

Then, the effect of VER and VEB on the innovation quality (green innovation) of textile enterprises is tested by using k-neighborhood 1:4 matching.

**Figure 12:**
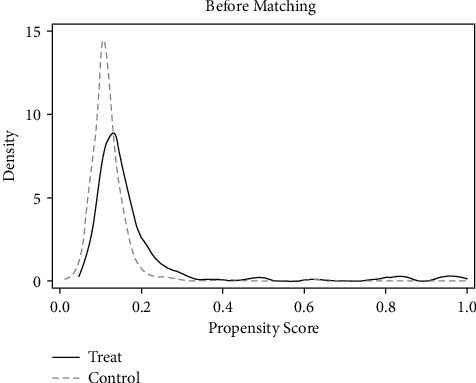
VER density function before matching.

**Figure 13:**
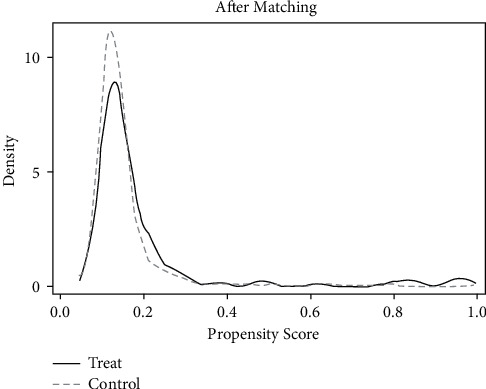
VER density function after matching.

**Figure 14:**
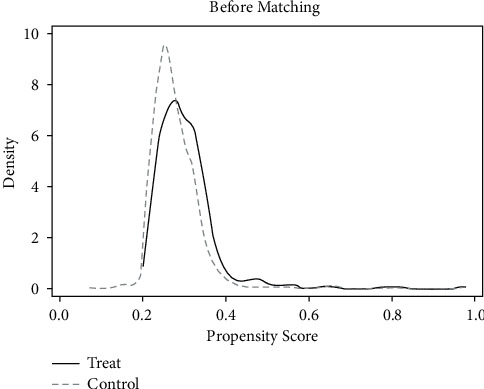
VEB density function before matching.

**Figure 15:**
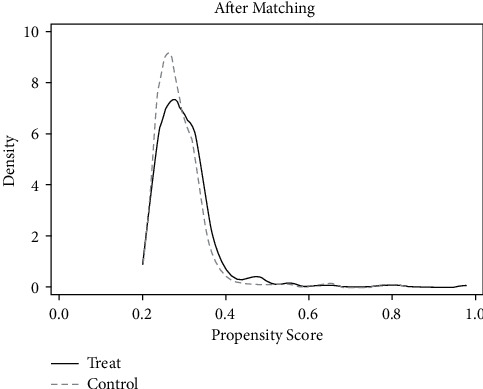
VEB density function after matching.

As mentioned above, the common support area and balance of matching samples were tested first. It can be seen from Tables 8 and 9 and Figures 12–15 that there are few lost samples and the matching effect is satisfied.

**Figure 16:**
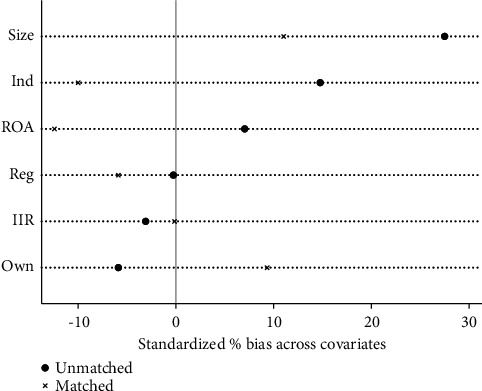
VER matching validity test.

**Figure 17:**
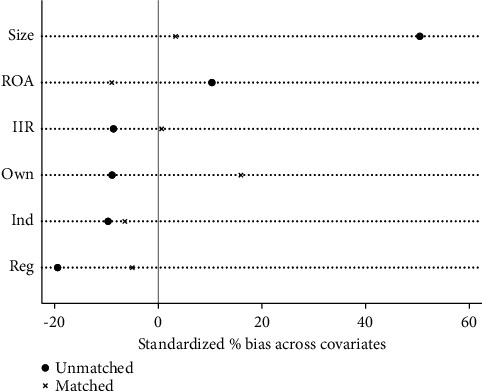
VEB matching validity test.

It can be seen from Tables 10 and 11 that both matching samples of VER and VEB have passed the balance test. Figures 16 and 17 visually show the improvement of sample bias after matching.

According to the model 1, to measure the effect of VER/VEB on innovation quality (green innovation) of textile enterprises, it can be seen from Tables 12 and 13 that implementing VER and VEB indeed can effectively promote the innovation quality (green innovation) of textile enterprises. Comparatively, VEB (the T-stat value of ATT is 5.52) has a more significant promoting effect on textile enterprises' innovation quality than VER (the T-stat value of ATT is 4.89). In other words, when Chinese textile enterprises voluntarily follow environmental regulations and implement environmental behaviors, the innovation quality of enterprises, especially green innovation, can be effectively promoted. It is also the original intention of environmental regulation.

Although the results of different matching methods are slightly different, if the conclusions drawn by different methods are similar, the matching results are robust [32]. The results of robustness test by different matching methods are consistent with the above results. Due to space limitation, the results of robustness test will not be presented in detail here.

## 4. Conclusion and Discussion

Based on the grounded theory, the paper establishes a comprehensive and specific theoretical framework for the motivation and influence path of enterprises' VEB; furthermore the PSM method is taken to verify the model, which can provide some references and enlightenment to the management practice of enterprises and governments, respectively.

To the enterprise, the implementation of VEB is not only to follow the external policies and regulations, but also to promote its industry position and pursue the excellence. Take the environment protection as enterprise strategy, and earnestly implement it in the daily operation, so as to improve enterprise competitiveness for the future sustainable development [33]. At the same time, to be specific, complying with VER such as ISO14001 can help enterprises to sort out their VEB and standardize it systematically. Voluntary environmental protection product certification is helpful to externalize the positive effect of VEB of enterprises to the market and consumers and form the overall green development of the supply chain system. Most importantly, either following VER or implementing VEB can effectively increase the innovation input and then actively improve the quality of innovation of textile enterprises.

To the government, first of all, it is better for the government to appropriately guide the VER, in addition to the traditional command-based and market-based environmental regulations. Although the enterprise voluntarily follows, if the government can provide some financial support and rewards [34], it can form a positive encouraging effect on the enterprises. Secondly, in terms of the environmental policy, in addition to the top national level policy, it can form more complete and targeted policy with industry level or regional level [35]. For example, we analyze the clothing industry and textile industry on the expression of VEB which will make a huge difference. In the aspect of green textiles, the textile industry has more practice of voluntary certification, relative to other industries. Therefore, more targeted guidance can improve the policy effectiveness in segmented areas [36].

At last, as all data sources in this paper are collected manually, there may be some errors, which is the biggest limitation of the study.

## Figures and Tables

**Table 1:** Open coding.

No.	Category	Example of original material	Source
1	Environmental policy	The company earnestly practices the development concept that “lucid waters and lush mountains are golden mountains and silver mountains”	NO.12
2	Position in industry	The very few enterprises in the spandex industry can produce this kind of products	NO.14
3	Honor	Awarded as “regional green enterprise,” “Henan green enterprise,” and other honorary titles	NO.3
4	Development strategy	Combine the company's development strategy with corporate social responsibility, and combine the development planning and practice of social responsibility with the company's own development goals	NO.6
5	Quality requirement	We have developed the treatment technology with independent patent, invested more than 110 million yuan, and implemented a number of technical transformation projects, so that the exhaust emission of the process is far below the national standard	NO.4
6	Regulation	Establish and improve emergency response plans for environmental emergencies, and formulate environmental self-monitoring plans	NO.1
7	Organizational structure	The top management assumes the responsibility for environmental protection and sets up green positions to implement green management of the enterprise	NO.2
8	Capital	Investment in environmental protection has increased year by year	NO.23
9	Technology	Research and develop technology on new environmental protection and energy saving, such as half-tank dyeing, reclaimed water reuse, and so on	NO.7
10	Supply chain	The company preferentially selects the dye and auxiliary suppliers that have passed the European environmental protection certification	NO.8
11	Standard	Voluntary product certification: STANDARD100byOEKO-TEX; T/CCFA 02007-2008; FSC, FSC-STD-40-004 V3-0	NO.21 NO.22
12	Cleaner production	Adhere to the environmental policy of “environmental factory, compliance, full participation, cleaner production, pollution reduction and efficiency, sustainable development”	NO.18
13	Environment protection	Production of safe and healthy textile products, leading the positive energy of environmental protection and sustainable development	NO.13
14	Transformation and upgrading	The company actively plays the role of a platform for scientific and technological innovation, stimulates the intelligence of scientific researchers, and promotes the industrial transformation and upgrading of the company	NO.4
15	Sustainable development	The company continues to deepen the concept of sustainable development within the enterprise and actively integrates environmental responsibility and social responsibility into the internal management system of the enterprise	NO.9
16	Circular economy	We will continue to promote clean production and a circular economy and strive to improve the comprehensive utilization of resources	NO.11

**Table 2:** Axial coding.

No.	Main category	Open category	Category connotation
1	External factor	Environmental policy	Environmental policy is an important external factor that drives enterprises to carry out VEB
		Position in industry	Companies are also more motivated to be proactive about environmental behavior if they are in a leading position in the industry

2	Internal factor	Honor	The honor that an enterprise can obtain in the field of environmental protection also promotes its VEB
		Development strategy	If environmental protection is regarded as an important aspect of corporate strategy, VEB is an important way to implement the strategy
		Quality requirement	The quality requirements of enterprises will drive enterprises not only to meet the minimum environmental requirements, but also to constantly improve

3	VEB	Regulation	By establishing internal environmental protection regulations, the VEB are regulated
		Organizational structure	Ensure the effective implementation of VEB by establishing environmental protection departments (e.g., environmental protection committee) within the enterprise
		Capital	Continuously increase the investment in environmental protection to ensure that VEB get sufficient financial support
		Technology	A series of technical transformation projects such as reclaimed water reuse and half-cylinder dyeing are adopted to improve the environmental protection capability of enterprises
		Supply chain	When selecting suppliers, enterprises will consider whether the supplier has environmental protection certification or the products have environmental protection

4	VER	Environmental management certification	ISO14001
		Product certification	GRS 4.0 RVA, STANDARD100byOEKO-TEX, FSC-STD-40-004 V3-0, T/CCFA 02007-2008

5	Performance		Whether VEB of enterprises meets the expectations or needs to be measured by environmental performance
	Direct	Cleaner production	For manufacturing textile enterprises, cleaner production is the first step to connect VEB with green performance
		Environment protection	Environmental protection is the direct target of enterprises' VEB
		Transformation and upgrading	As a traditional manufacturing industry, constantly adapting to the development of the times, the pursuit of transformation and upgrading is also one of the ultimate goals
	Indirect	Sustainable development	Green development is sustainable, and VEB of enterprises can help them achieve sustainable development
		Circular economy	With increasingly scarce resources, enterprises through VEB can achieve energy saving and consumption reduction of circular economy

**Table 3:** Selective coding.

Relational structure	Connotation of relational structure
Motivation (External/Internal) ⟶ VEB/VER	Internal and external factors such as policy, position in industry, honor, development strategy, and quality requirements are all important factors influencing the implementation of VEB and compliance with VER
VEB ⟶ VER/VER ⟶ VEB	Enterprises' implementation of VEB and compliance with VER are mutually reinforcing. Enterprises with active implementation of VEB are more willing to comply with VER (such as voluntary environmental product certification). Companies that have followed VER (such as ISO14001) will be more active in implementing VEB
VEB/VER ⟶ Performance	The effect of implementing VEB and complying with VER will be reflected in the environmental performance of enterprises and the sustainability of their future development
External Motivation⟶VEB/VER⟶Performance	The external motivation such as policy environment and the position in industry of enterprises will affect the implementation of VEB and compliance with VER, which will be reflected in the environmental performance and economic effect of enterprises
Internal Motivation ⟶ VEB/VER ⟶ Performance	The internal motivation such as corporate honor, development strategy, and quality requirement will affect the implementation of VEB and compliance with VER and then be reflected in the environmental performance and economic effect of the enterprise
Motivation ⟶ VEB/VER ⟶ Performance	Internal and external factors interact with each other to influence the implementation of VEB and compliance with VER, which are reflected in the environmental performance and economic effects of enterprises

**Table 4:** VEB vs. VER.

	VEB (no. of text)	VER (ISO14001)
VEB (no. of text)	1	
VER (ISO14001)	0.201	1

**Table 5:** PSM results.

Psmatch2:Treatment	Psmatch2: common support		Total
Assignment	Off support	On support	
Untreated	0	1,190	1,190
Treated	1	474	475
Total	1	1,664	1,665

**Table 6:** Balance test before and after covariable matching.

Variable	Unmatched	Mean		%bias	%reduct bias	*t*-test		*V*(*T*)/*V*(*C*)
	Matched	Treated	Control			*t*	*p* > *t*	
Ind	U	2.1747	2.0311	14.8		2.72	0.007	1
	M	2.173	2.2737	−10.3	29.9	−1.53	0.125	0.86

Own	U	0.76632	0.79076	−5.9		−1.09	0.274	.
	M	0.76582	0.727	9.3	−58.8	1.37	0.17	.

Reg	U	5.7747	5.7874	−0.3		−0.05	0.961	0.98
	M	5.7848	6.0593	−5.7	−2068.4	−0.87	0.383	0.95

Size	U	4.90*E*+09	2.70*E*+09	27.5		5.27	0	1.42^*∗*^
	M	4.60*E*+09	3.80*E*+09	10.1	63.3	1.77	0.077	0.85

IIR	U	0.12716	0.15176	−3.1		−0.48	0.628	0.06^*∗*^
	M	0.12686	0.12862	−0.2	92.9	−0.1	0.923	0.79^*∗*^

ROA	U	0.05293	0.04712	7		1.21	0.226	0.49^*∗*^
	M	0.05299	0.06321	−12.4	−76	−1.85	0.064	0.45^*∗*^

**Table 7:** The treatment effect of PSM (VER, innovation input).

Variable	Sample	Treated	Controls	Difference	S.E.	*T*-stat
Rdexp	Unmatched	52062112.4	23180778.3	28881334.1	3778724.08	7.64
	ATT	50154694.9	35042009	15112685.9	4712594.03	3.21

**Table 8:** PSM results (VER).

Psmatch2:	Psmatch2:Common support	Total
Treatment assignment	On support	
Untreated	1,190	1,190
Treated	475	475
Total	1,665	1,665

**Table 9:** PSM results (VEB).

Psmatch2:	Psmatch2: Common support		Total
Treatment assignment	Off support	On support	
Untreated	0	1,448	1,448
Treated	1	216	217
Total	1	1,664	1,665

**Table 10:** Balance test before and after covariable matching (VER).

Variable	Unmatched	Mean		%bias	%reduct	*t*-test		*V*(*T*)/*V*(*C*)
	Matched	Treated	Control		bias	*t*	*p* > *t*	
*Ind*	U	2.1747	2.0311	14.8		2.72	0.007	1
	M	2.1747	2.272	−10	32.3	−1.48	0.138	0.86

*Own*	U	0.76632	0.79076	−5.9		−1.09	0.274	
	M	0.76632	0.72758	9.3	−58.5	1.37	0.17	

*Reg*	U	5.7747	5.7874	−0.3		−0.05	0.961	0.98
	M	5.7747	6.0576	−5.9	−2134.5	−0.9	0.368	0.95

*Size*	U	4.90*E*+09	2.70*E*+09	27.5		5.27	0	1.42^*∗*^
	M	4.90*E*+09	4.00*E*+09	11	59.9	1.6	0.11	1.09

*IIR*	U	0.12716	0.15176	−3.1		−0.48	0.628	0.06^*∗*^
	M	0.12716	0.12831	−0.1	95.3	−0.06	0.95	0.79^*∗*^

*ROA*	U	0.05293	0.04712	7		1.21	0.226	0.49^*∗*^
	M	0.05293	0.06317	−12.4	−76.5	−1.86	0.063	0.45^*∗*^

**Table 11:** Balance test before and after covariable matching (VEB).

Variable	Unmatched	Mean		%bias	%reduct	*t*-test		*V*(*T*)/*V*(*C*)
	Matched	Treated	Control		bias	*t*	*p* > *t*	
*Ind*	U	1.9908	2.0843	−9.7		−1.32	0.188	0.95
	M	1.9861	2.0485	−6.5	33.3	−0.69	0.488	1.09

*Own*	U	0.75115	0.78867	−8.9		−1.25	0.211	.
	M	0.75	0.68295	15.9	−78.7	1.55	0.123	.

*Reg*	U	4.9816	5.904	−19.4		−2.64	0.008	0.94
	M	5	5.2383	−5	74.2	−0.53	0.6	0.97

*Size*	U	8.00*E*+09	2.60*E*+09	50.5		10.04	0	5.83^*∗*^
	M	7.50*E*+09	7.10*E*+09	3.3	93.4	0.28	0.779	0.64^*∗*^

*IIR*	U	0.09037	0.15289	−8.6		−0.92	0.359	0.05^*∗*^
	M	0.08955	0.08489	0.6	92.6	0.22	0.826	0.92

*ROA*	U	0.05615	0.04768	10.3		1.32	0.187	0.64^*∗*^
	M	0.05631	0.06367	−9	13.2	−0.84	0.404	0.46^*∗*^

**Table 12:** The treatment effect of PSM (VER, innovation quality).

Variable	Sample	Treated	Controls	Difference	S.E.	*T*-stat
Green	Unmatched	0.953684211	0.36302521	0.590659	0.074602684	7.92
	ATT	0.953684211	0.462365003	0.491319207	0.10055091	4.89

**Table 13:** The treatment effect of PSM (VEB, innovation quality).

Variable	Sample	Treated	Controls	Difference	S.E.	*T*-stat
Green	Unmatched	1.29953917	0.416436464	0.883102706	0.09960144	8.87
	ATT	1.30555556	0.420138889	0.885416667	0.160373379	5.52

## Data Availability

The data that support the findings of this study are available on request from the corresponding author.
